# Perceived Sound Quality of Hearing Aids With Varying Placements of Microphone and Receiver

**DOI:** 10.1044/2022_AJA-22-00061

**Published:** 2022-12-29

**Authors:** Michael A. Stone, Melanie Lough, Volker Kühnel, Anna E. Biggins, Helen Whiston, Harvey Dillon

**Affiliations:** a Manchester Centre for Audiology and Deafness, The University of Manchester, United Kingdom; b Hearing Device Research Centre, Hearing Health, National Institute of Health Research Manchester Biomedical Research Centre, United Kingdom; c Sonova AG, Staefa, Switzerland; d Department of Linguistics, Macquarie University, Sydney, New South Wales, Australia

## Abstract

**Purpose:**

Perceived sound quality was variously compared between either no aiding or aiding with three models of hearing aid that varied the microphone position around the pinna, depth of the receiver in the auditory meatus, degree of meatal occlusion, and processing sophistication. The hearing aids were modern designs and commercially available at the time of testing.

**Method:**

Binaural recordings of multichannel spatially separated speech and music excerpts were made in a manikin, either open ear or aided. Recordings were presented offline over wide-bandwidth, high-quality insert earphones. Participants listened to pairs of the recordings and made preference ratings both by clarity and externality (a proxy for “spaciousness”). Two separate groups of adults were tested, 20 with audiometrically normal hearing (NH) and 20 with mild-to-moderate sensorineural hearing loss (hearing impaired [HI]).

**Results:**

For ratings of speech clarity, the NH group expressed no preference between the open ear and a deeply inserted occluding aid, both of which were preferred to a low-pass filtered output of the same aid. For the music signal, a small preference emerged for the open-ear recording over that of the aid. For the HI group, clarity of the deeply inserted aid was similar to in-the-ear and behind-the-ear devices for speech, but worse for music. Ratings of spaciousness produced no clear result in either group, which can be attributed to study limitations and/or participant factors.

**Conclusion:**

Based on clarity, a wide bandwidth, particularly to beyond 5 kHz generally and below 300 Hz for music, is desirable, independent of hearing aid design.

The selection of a hearing aid (HA) for a patient, apart from the ability to provide gain sufficient to remediate the hearing loss, is a compromise between several competing factors (Jorgensen & Novak, 2020; Picou, 2020). One such choice is in the form of processing, analog, or digital. Since the millennium, digital processing has largely replaced analog. The success of digital processing has been attributed to its offering of flexibility, capabilities, and repeatability, which was largely unachievable in analog HAs (Levitt, 2007). This success was not achieved without compromises, such as a finite audio bandwidth, higher power consumption (translating to shorter battery life), and markedly increased delay of the audio signal through the HA (Dillon et al., 2003; Frye, 2001; Kates & Arehart, 2005), until recently (Balling et al., 2020). Some of these compromises have the potential to degrade audio quality compared to that which was already achievable in a well-designed analog HA (Killion & Tillman, 1982). Indeed, comparisons of satisfaction from users of either analog or early digital aids showed little difference between the two (Bille et al., 1999; Parving, 2003; Wood & Lutman, 2004).

A further factor considered in HA selection is in the style (behind-the-ear [BTE], in-the-canal, etc.). In order to address some of these competing factors, most digital HAs are a daily-wear device that is removed regularly, such as for periods of sleep or for activity in hostile environments, such as swimming. Miniaturization of components, as well as custom digital processing, produces devices that typically can last for 1 week without a battery change. In contrast, pursuit of an invisible HA has led to the design of devices that are ultraminiature or even surgically implanted. However, the power consumption of the current generations of digital processing makes the technology unsuitable for implantation since regular recharging of the power source is required. The lower power consumption and lower complexity of analog processing can circumvent this to create niche products. There exist many designs for ultralow-power audio processor components (Medeiros et al., 2013; Park et al., 2020; Wang et al., 2014; Yang et al., 2015) that could be suitable for either surgical implantation or semipermanent wear with a suitable high-capacity battery.

One such niche analog product is the Phonak “Lyric,” a device designed to be positioned to within 4 mm of the eardrum powered by a single nonrechargeable and nonreplaceable battery. When fitted correctly, it should occlude the ear canal without any visible gaps in the soft flanges of the device (Phonak, 2016); it is unvented. We refer to this device as a “DIAA,” a deeply inserted analog aid.

There are several reasons why a difference in sound quality is expected between a DIAA and more conventional styles of HA:

*1. The effect of the typically shorter processing delay between the microphone and loudspeaker (receiver) in an analog, rather than a digital, HA.*

There are multiple sound paths to the cochlea, direct, such as via vents and bone conduction, and indirect, through the processing delay of the HA. Where the sounds combine from the different sound paths, if the sounds are of similar level, the frequency response becomes lumpy, a feature referred to as “comb filtering” due to the shape of the response (Stone et al., 2008). For delays longer than 1 ms, this can lead to comb filtering at frequencies below 1 kHz, where little amplification is required for low degrees of hearing loss; hence, the direct and indirect sound paths are similar in level. Figure 1 in Balling et al. (2020) reports delays for anonymized modern digital HAs in the range of 5–8 ms for frequencies below 1 kHz and 2–8 ms between 4 and 8 kHz. For such short delays, the perceived sound acquires a change in timbre rather than any percept of an echo.

Relative to a digital HA, the shorter processing delay introduced by the DIAA, around 200 μs (Phonak, personal correspondence), as well as its “earplug effect” should result in a negligible comb-filtering effect. A recent digital HA, the Widex PureSound, also offers sub–1-ms delay (Balling et al., 2020) but was not available at the time of this study and is more open in its fitting in the meatus.

*2. Deeper microphone placement in the meatus will more accurately sample the sound field as presented to the eardrum than would a microphone placed on the pinna or in the concha.*

When in situ, the Lyric microphone will be at a recommended 4–16 mm within the external meatus (Phonak, 2016). As such, the frequency response at the microphone is expected to differ from that in traditional in-the-ear (ITE) and BTE styles of HA (Bentler & Pavlovic, 1989), where the microphone is situated in the concha or on top of the pinna, respectively. The meatal positioning of the microphone gives the wearer more access to the monaural spectral cues arising from the natural sound-modifying abilities of the head, pinna, and concha that, theoretically, can help with sound localization and the perception of externality (Boyd et al., 2012). In practice, such improvements have largely been demonstrated in people with normal hearing (NH); people with hearing loss tend to be less sensitive (and more variable in their responses) to the spectral cues that externalize sound (Boyd et al., 2012; Ohl et al., 2010). However, studies that have examined the effect of HA style on localization ability in hearing-impaired (HI) subjects (e.g., Van den Bogaert et al., 2011) have conducted their experiments with more conventional HAs, not a DIAA. A comparison of a DIAA against an ITE device could indicate whether this difference in placement has an effect on localization or externalization.

*3. The location of the receiver in a DIAA potentially produces a wider frequency response.*

Miniature acoustic transducers generally cannot deliver high sound levels at low frequencies. The smaller acoustic volume of the meatus being driven by a DIAA will permit the delivery of useful levels of low-frequency sound (typically enabling delivery below 400 Hz; Killion et al., 2016) when compared to a more conventional HA (e.g., with venting).

*4. A deep insertion depth should reduce the “occlusion effect.”*

The *occlusion effect* (Killion et al., 1988; von Békésy, 1960) is where listeners perceive an increase in the low-frequency content of a self-produced, bone-conducted sound when their ear canal is blocked in some way (e.g., by an earplug or ear mold). Deeper insertion produces a lower degree of occlusion (Stenfelt & Reinfeldt, 2007). The open-fitting approach (Smith et al., 2008), made possible by digital feedback suppression, also reduces the occlusion effect, permitting low-frequency information to arrive at the cochlea via the air leakage path around the meatal fitting, an alternative method of extending the low-frequency response (rather than using a smaller closed volume, as in 3 above). This extension is at the expense of introducing the timbre changes associated with comb filtering.

The purpose of this study was, therefore, to compare binaural aiding with a DIAA with that of two types of daily-wear digital HAs in terms of perceived clarity and spaciousness (two of seven dimensions of sound quality identified by Gabrielsson, 1979). In order to provide a “real-world” comparison, we used clinically available devices and their associated fitting software, rather than research devices or simulators. Consequently, it was not possible to perform a comparison between otherwise “twinned” HA processing, that is, identical in every respect except for the processing implementation (e.g., Brennan et al., 2014). Our aim was to provide better insight as to the impact different styles of commercially available HA have on the perceived sound qualities most likely to be affected by the acoustic consequences of adopting each style.

The study reported here therefore additionally provides a chance for update on the generally disappointing findings of the earlier studies comparing analog versus digital HAs, mentioned above, but with some unavoidable constraints. With the HA market offering very few analog aids and dominated by digital HAs, there are no recent replications of those studies using more modern (post 2004) and presumably improved devices. With other changes in technology, such as more open meatal fittings, replicating exactly these early studies is unlikely to be possible.

There have been previous comparisons of the quality of audio bandwidth in NH listeners (e.g., Moore & Tan, 2003) or HA bandwidth with HI listeners (Brennan et al., 2014; Franks, 1982; Füllgrabe et al., 2010; Ricketts et al., 2008). Since the commercial HAs to be tested unavoidably covaried in several parameters, then apart from using an HI group to test HAs, we also included an NH group. With this group, we could compare the basic acoustic “transparency” of the DIAA against the fidelity of an unaided, open-ear recording. This NH group approach, by permitting a closer match to desired insertion gain required to imitate the open ear (i.e., acoustic transparency), also reduced the heterogeneity of response that arises with the varying degrees of hearing impairment found in an HI group. The influence of the remaining main variables, bandwidth and processing of dynamics, should then be greater in the NH group results, offering clearer insight into their relative contribution and so providing wider insight into the results from an HI group.

## Method

### Participants

Participants were recruited from a variety of non–National Health Service sources. Ninety-nine people were screened, and 40 were included based on the criteria shown in Table 1. The recruitment of participants and test methods employed were approved by The University of Manchester Research Ethics Committee (Reference Nos. 2018-4622-6902 and 2019-4644-11260).

**Table 1.** Selection criteria for both groups of test subjects.

NH group (*n* = 20)	HI group (*n* = 20)
Aged 18–35 years	Aged 45–80 years
Pure-tone hearing thresholds between 0.125 and 12.5 kHz were ≤ 15 dB HL	Pure-tone hearing thresholds at 1–6 kHz were ±10 dB of standard N2 or N3 audiograms bilaterally (allowing for one outlier on each side)
	Thresholds between 0.125 and 0.5 kHz were ≤ 45 dB HL
**Both groups**	
No previous mastoidectomy	
Ear canals not occluded with wax	
English-speaking (as a first language) from birth	
A score of ≤ 7 on the 6CIT cognitive screen (Abdel-Aziz & Larner, 2015)	
Average air–bone gap at 0.5, 1, and 2 kHz ≤ 10 dB bilaterally	
No asymmetry in hearing thresholds (defined as a difference in right and left AC or BC thresholds of ≥ 20 dB at two or more frequencies)	
Ear canal volume and middle ear pressure within normal limits (0.6–2.5 cm^3^ and −50 to +50 daPa, respectively; British Society of Audiology, 2014) and compliance of ≥ 0.3 cm^3^, as demonstrated with 226-Hz tympanometry	

*Note.* NH = normal hearing; HI = hearing impaired; 6CIT = six-item cognitive impairment test; AC = air conduction; BC = bone conduction.

The equipment used for screening potential participants comprised a GSI Pello audiometer and GSI 39 Auto Tymp (tympanometer). Air-conduction audiometry was conducted using RadioEar DD450 circumaural headphones at standard audiometric frequencies, as well as 125 and 12500 Hz, and bone-conduction testing was performed with a RadioEar B-81 vibrator. Bone-conduction masking, if required, was delivered through RadioEar IP30 insert earphones.

The NH group comprised 16 women and four men with a mean age of 23.9 years (range: 18–32). All had scores of ≤ 4 on the six-item cognitive impairment test (6CIT) cognitive screen (Abdel-Aziz & Larner, 2015); < 8 is considered a “pass.” No participant in the NH group reported any subjective hearing difficulties, and tympanometry was within normal limits (ear canal volume within 0.6–2.5 cm^3^, middle ear pressure from −50 to +50 daPa, and middle ear compliance within 0.3–1.6 cm^3^; British Society of Audiology, 2014) bilaterally for all participants.

**Figure 1.**
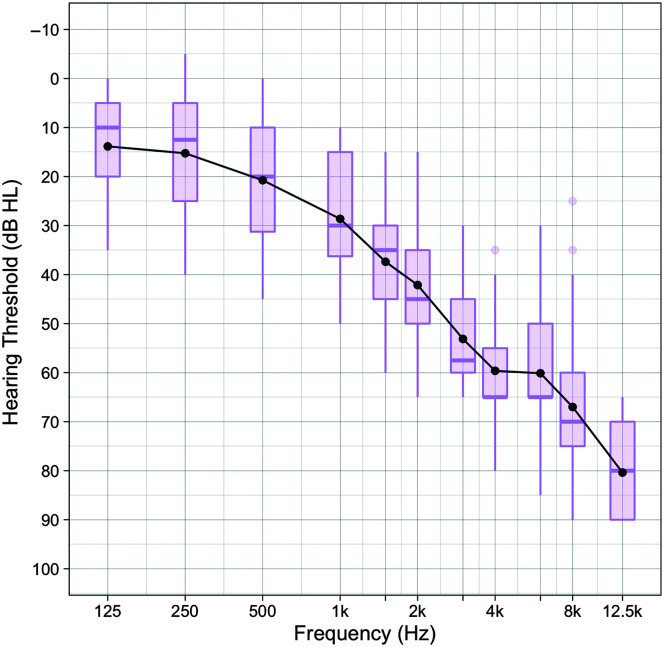
Audiogram for the hearing-impaired group (total *n* = 20, 40 ears), displaying mean hearing thresholds (black circles), as well as median pure-tone hearing thresholds (bars) and interquartile ranges (box plots). Whiskers extend from the smallest to the largest data points within 1.5 times the interquartile range below the 25th or above the 75th percentile, respectively.

The HI group comprised six women and 14 men with a mean age of 64.4 years (range: 55–78). All had 6CIT scores of ≤ 4. Nineteen participants reported subjective hearing difficulties, although only 12 were regular HA users. Tympanometry was within normal limits (British Society of Audiology, 2014) in 12 participants. Of the remaining eight, two had bilateral hypermobile middle ear systems (middle ear compliance exceeding 1.6 cm^3^), and six had unilateral middle ear hypermobility. Middle ear pressures and ear canal volumes were within normal limits for these participants, and no conductive element was observed on their audiograms. The median pure-tone hearing thresholds and the respective interquartile ranges for the HI group (40 ears) are displayed in Figure 1. The average air–bone gap at 0.5, 1, and 2 kHz was −1.5 dB (range: −11.7 to 6.7). No response was recorded at 12.5 kHz for 13 ears due to the hearing threshold exceeding the maximum stimulus level of the audiometer (90 dB HL).

HI participants were further split into two subgroups to ensure that the delivery of the (audio) test material was within, or close to, clinical tolerances for insertion gain appropriate to their losses. This decision was based on whether their mean threshold at 1–6 kHz (in the better ear) was closer to the mean threshold of the N2 or N3 standard audiograms (Bisgaard et al., 2010). Eleven participants were assigned to the N2 subgroup (two women and nine men; *M*_age_ = 63.1 years); nine were assigned to the N3 subgroup (four women and five men; *M*_age_ = 66.0 years).

### The HAs Compared

Three HAs were selected for comparison as belonging to the premium end of the market at the time that the experiment was designed:

1. the DIAA, a Phonak “Lyric3” (size XS);
2. an ITE aid, the Phonak Virto B-Titanium, referred to hereafter as “ITE”; and
3. a BTE aid with a receiver-in-the-canal, the Phonak Audéo B-10, referred to hereafter as “RIC.”

The DIAA employed a single-channel, very fast-acting limiter function at high output levels (85 dB SPL at 2 kHz, attack = 2 ms, release = 10 ms). The digital aids both employed 20 channels of wide dynamic range compression, with speed automatically selected according to program type. Fast compression (attack and release time of 10 and 60 ms, respectively) was used for speech and slow compression (attack and release time of 1 and 8 s, respectively) for music. Further details of the digital compression are given in Chen et al. (2021). Measured using 0.1-ms-duration acoustic impulses, both digital HAs showed a constant across-frequency processing delay of around 6.5 ms.

All acoustic comparisons of the aids were performed by making recordings on a KEMAR (Burkhard & Sachs, 1975) head and torso simulator. The head was fitted with small silicone–rubber pinnae of low hardness (35 Shore OO). Each meatus was terminated by an “IEC 711” coupler (EN60318-4, 2010) and a Brüel & Kjær 4192 microphone. The microphone signal was conditioned by a Brüel & Kjær 2669-L powered by a GRAS 12AA power module (incorporating a stepped-gain amplifier). The coupler microphone sensitivities were calibrated before all recordings using a Brüel & Kjær 4231. While the DIAA and RIC fitted easily into the cylindrical meatus of the coupler due to the deformability of their flanges, the ITE, being a hard shell, was custom-made to fit the meatus.

### HA Programming

The programming of the aids was performed by simulating three separate 70-year-old male “clients” in Phonak Target 5.3.3 fitting software. The clients differed solely in their audiogram. These were as follows:

1. a standard N2 audiogram (Bisgaard et al., 2010),
2. a standard N3 audiogram (Bisgaard et al., 2010), and
3. a mild low-frequency loss. This audiometric configuration was used as it resulted in a near-flat frequency response from the DIAA device with a gain exceeding 0 dB. The elevation of gain was necessary so as to preserve a good output signal-to-noise ratio in the recordings (the “noise” being the internal noise of the device, i.e., electrical). On replay to the NH group only, this elevated gain was removed.

All three types of HA devices were separately programmed to Clients 1 and 2 (depending on the sound recording to be made). Only the DIAAs were programmed for Client 3. For each client, the audiogram was the same for the right and left ears. The parameters selected in the fitting software are given in Table 2. The size of vent (ITE) and dome-type choice (RIC) were in keeping with the recommendations in the fitting software and were appropriate for the degree of hearing loss in the study sample.

**Table 2.** The parameters selected in the fitting software for each of the three hearing devices.

Variable	Phonak Lyric3 (DIAA)	Phonak Virto B-Titanium B90 M (ITE)	Phonak Audéo B-10 (RIC)
Size/acoustic parameters	XS^a^	The molded shell was custom-made for KEMAR ears 1.7-mm vent	B90 0xS receiver Large closed domes^b^ (enabled N3 gains without feedback)
Measured insertion depth	10 mm		
Achieved insertion depth	10 mm^c^		
Gain prescription	Proprietary^d^	NAL-NL2 (100%)	NAL-NL2 (100%)
Listening programs	“Default precalculation”	AutoSense OS only	AutoSense OS only
Blending		Fast	Fast
Frequency compression (sound recover)		Off	Off
Volume control	Range reduced as far as possible	Off	Off
Microphone modes			Default
Feedback suppression	N/A	Off	Off

*Note.* DIAA = deeply inserted analog aid device; ITE = in-the-ear hearing aid device; RIC = receiver-in-the-canal hearing aid device.

a This size enabled full occlusion of the KEMAR meatus without visible distortion of the foam flanges.

b “Closed” is the manufacturer's term for this dome. It comprises a single flange, with two small vents.

c Based on measurements taken in KEMAR, with the devices in situ. It was not possible to insert the devices further into the ears of the KEMAR due to the physical design of the coupler.

d NAL-NL2 was not an available option, but adjustments were later made to meet NAL-NL2 targets.

**Figure 2.**
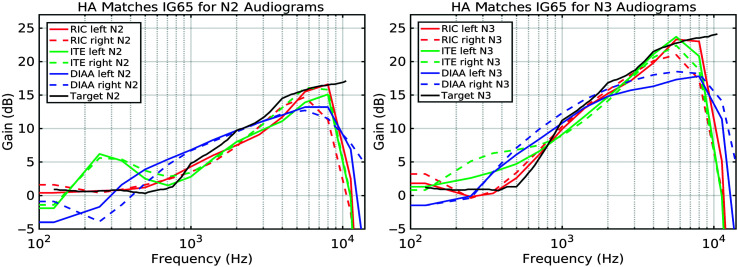
Insertion gain matches to NAL-NL2 for the N2 audiogram (left panel) and N3 audiogram (right panel). HA = hearing aid; RIC = receiver-in-the-canal hearing aid device; ITE = in-the-ear hearing aid device; DIAA = deeply inserted analog aid device.

**Figure 3.**
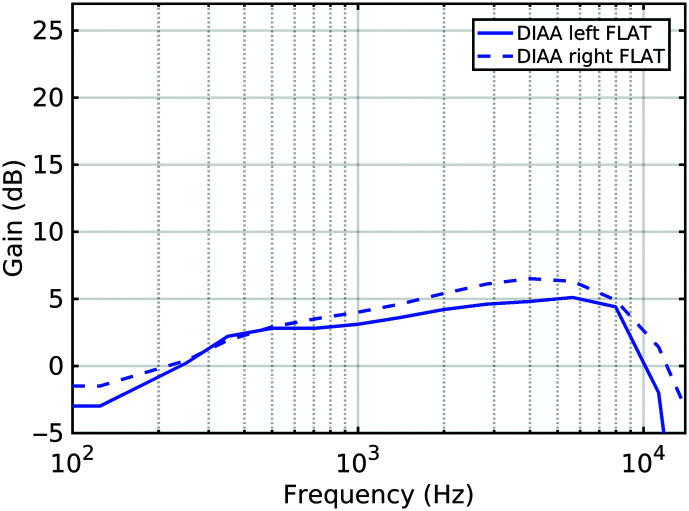
Insertion gain measurements in KEMAR with the deeply inserted analog aid (DIAA) programmed for as near-flat response as possible.

Small changes were made in the settings away from the default “first fits” for all devices in order to obtain better matches to the prescribed insertion response, thus simulating a clinical fitting. Real-ear insertion gain (REIG) was measured by making open-ear and aided-ear recordings in the KEMAR manikin of the International Speech Test Signal (ISTS; Holube et al., 2010) being replayed from a frontal loudspeaker at 65 dB SPL. Off-line, a transfer response was generated between the two recordings. For the digital devices, any necessary within-channel gain adjustments were made for all input levels (i.e., 50, 65, and 80 dB) within that channel, leaving the dynamics unaltered. For the DIAA, which had far less flexibility of software control than for the digital devices, changes had to be made to both the “low-frequency cut” and “volume” (but not the “slope” control). The obtained matches to the NAL-NL2 target for the N2 audiogram are displayed in the left panel of Figure 2 and for the N3 audiogram in the right panel. The near-flat elevated gain responses achieved for the DIAAs are shown in Figure 3.

**Figure 4.**
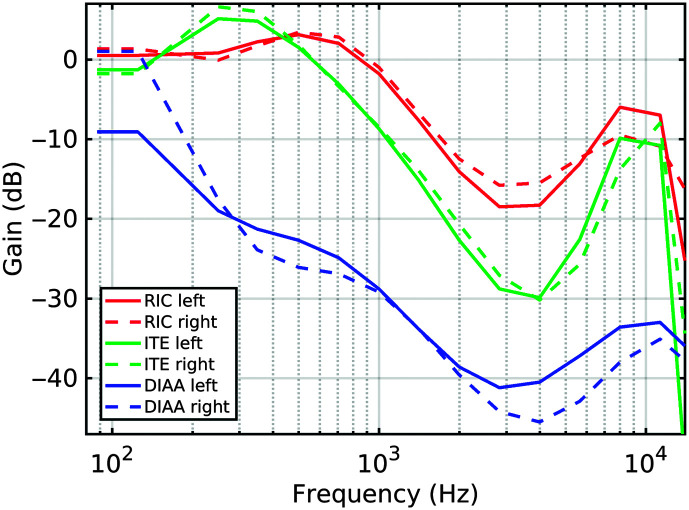
Occluded ear insertion gains (real-ear occluded gains) as a function of hearing aid: the “earplug effect.” RIC = receiver-in-the-canal hearing aid device; ITE = in-the-ear hearing aid device; DIAA = deeply inserted analog aid device.

The “earplug” effect of each device (real-ear occluded gain [REOG]) was measured with the same method as for the REIG, with the HAs in situ, but switched off, and also open ear (i.e., with the HAs removed), using the 70 dB SPL pink noise stimulus from the same frontal speaker. The transfer function between the two recordings defines the REOG. This helps with the interpretation of the later results. The responses are displayed in Figure 4. This shows the DIAA acting as an effective earplug to external sounds across a wide range of frequencies. We therefore expected the meatal sound to consist almost entirely of that which was processed by the HA. Conversely, the digital devices were acoustically near-transparent to direct-path sounds for frequencies up to about 1 kHz and acting as an earplug only at higher frequencies. Additionally, there is evidence of a resonance effect boosting transmission below 1 kHz in both devices due to the change in ear canal acoustics from the physical construction of the devices, principally the venting obtained (centered on 300 Hz for the ITE and 500 Hz for the RIC). The perceived sound from these devices when active therefore comprises near-equal levels of direct and HA-processed sound across part of the low-frequency range and the HA-processed sound only dominating at medium to high frequencies. Repeated measures of the REOG were taken; the DIAA exhibited more variation below 300 Hz than for the other two styles of HAs. The traces shown are representative of the range observed.

### Test Material

Two types of materials were prepared for recordings, speech and music. The speech material comprised a male voice counting down the digits from 10 to zero. The duration of the digit recordings was 7.1 s. The music material comprised a rendition of the jazz classic, “Fever,” by Chuck Brown. The chosen excerpt, of duration 7.3 s, included male vocal, finger clicks, electronic piano, drums, and some “stab” chords on the piano, so as to vary the frequency range, duration, and dynamics of the individual instruments.

**Figure 5.**
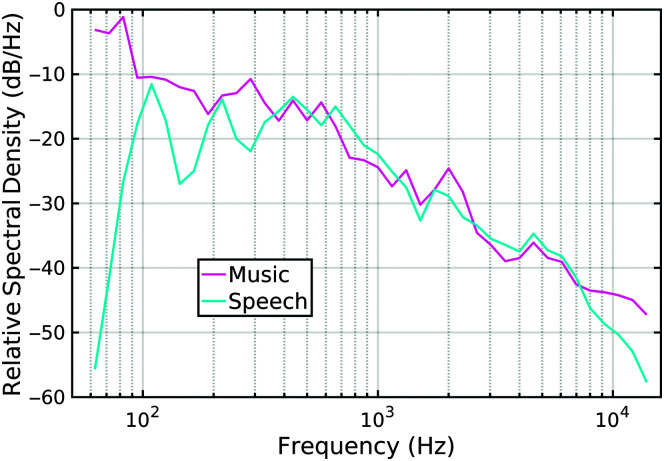
Comparison of the relative power spectral density (dB/Hz) of the music excerpt (magenta line) and the center channel talker (cyan line). The spectral densities have been smoothed in 1/10th-octave bands.

A comparison between the power spectral density of the music excerpt and the reference speech is given in Figure 5, as measured from the source files, but not accounting for presentation level. The two traces are offset so that the similar pattern of frequency content between 400 and 7000 Hz acts as a baseline, but the music has relatively more power than the speech both lower and higher in frequency than within this baseline span.

### Experiment Overview

The experiment was conducted in two phases. The first phase involved recording the HA outputs via the KEMAR manikin separately for the speech and music excerpts. The recordings were then processed to be suitable for delivery by insert earphones. In the second phase, the recordings were delivered to the participants over insert earphones during a test session. This permitted a blinded approach during testing with rapid switching between aid outputs in software rather than the participants swapping the wearing of physical devices.

### Sound Recordings

#### Loudspeaker Siting

All recordings took place in a carpeted listening room (4.9 × 3.5 × 2.8 m, length × width × height) with multiple absorbers distributed on the walls and ceiling of the room, resulting in a reverberation time (RT60) of 150 ms, relatively flat across 125–8000 Hz. The background noise level was less than 30 dBA SPL.

A Tannoy VXP 6 dual concentric, self-powered loudspeaker was placed directly ahead of the KEMAR head and torso at a distance of 1 m from the middle of the head. The KEMAR was placed near the middle of the room, equidistant from the long walls. KEF Q150 dual concentric, passive loudspeakers were placed at both +60° and −60° azimuth relative to the centerline of the dummy head, again at a distance of 1 m from the head. This positioning was as an extreme stereo pair (normal placement for stereo would be at ±30° off centerline). The Q150s, despite being “bookshelf” in size, have a good low-frequency response, −3 dB at 50 Hz, and so are suitable for the reproduction of both music and speech. The VXP 6 has a −3 dB response at 90 Hz, so it is more suitable for speech, rather than music, reproduction. All loudspeakers were on stands with the cone centerlines being 1.4 m off the ground, aligned with the centerlines of the KEMAR ears, as marked on the side of the head.

#### Replay and Recording Equipment Setup

Replay of the audio signals via the loudspeakers was under control of a PC running MATLAB (MathWorks) separate from the PC controlling the recordings. The sound card used for replay was a PreSonus Studio 26 USB interface, which could handle a maximum of four output channels.

The conditioned signals from the KEMAR-mounted meatal microphones were fed to a PreSonus V22SL two-channel USB sound card attached to another PC running MATLAB under Windows 7. The microphone gain, of +20 dB, was chosen so as to ensure use of the full electrical signal range of the sound card without digital clipping. All recordings were made with a resolution of 24 bits.

Calibration of the recording system involved measurement of the digital root-mean-square in the recorded WAV files corresponding to a known sound pressure level in the diffuse field (after correction for the diffuse field to eardrum transfer function for KEMAR). The left–right balance of level and frequency response in the recording system was checked by the use of a 70 dB SPL pink noise presented from the center loudspeaker, plotting the difference in the level between the two ears as a function of frequency. The KEMAR open-ear across-frequency difference was in the range of ±1.5 dB from below 100 Hz to 12 kHz.

For the speech material, the digits were presented through the center loudspeaker. The left-hand Q150 replayed a female competing talker, and the right-hand Q150 replayed a male competing talker. Both competing talkers were reading prose passages where pauses for breath had been removed by manual editing, but natural pauses between sentences had been preserved. Since the digital HAs used in this study had signal processing that adapted according to measured statistics of the signal (before settling into a particular listening mode, such as “speech in noise”), the desired speech passage for the experiment was embedded at the end of a much larger sound file. The precursor to this speech was the 60-s-duration ISTS replayed through the center loudspeaker at the same level as the target speech. Twenty-three seconds before the desired digits, the competing talkers were added through their respective loudspeakers. The duration of the digit recordings was 7.1 s. The replay level for the center channel was 60 dBA, while the side speakers were each at a level of −2 dB relative to this (total power of 63.5 dBA).

Prior to the replay of the music material, there was a continuous replay of nearly 120 s of three other music styles to ensure the stability of any automatic features in the HAs and also to provide some alternative genres, in case the jazz recording was not deemed suitable. The Q150s were calibrated for each to deliver 70 dBA at, but in the absence of, the dummy head. The VXP 6 was then not used.

#### Recording Conditions to Be Compared

Recordings were made in response to both speech and music stimuli in each of nine conditions. Two recording sessions were performed, once for the conditions to be used with the NH group and once for the conditions to be used with the HI group.

For the part of the experiment intended for the NH group, the conditions were as follows:

1.  open-ear KEMAR and
2.  DIAA calibrated so as to produce a near-flat insertion gain (as described in the HA Programming section above).

For the part intended for the HI group, the conditions were as follows:

3.  open-ear KEMAR,
4. & 5.  the DIAA programmed to compensate for the loss associated with either an N2 or an N3 audiogram (Bisgaard et al., 2010),
6. & 7.  the ITE also programmed to compensate for the losses described in 4 and 5 above, and
8. & 9.  the RIC programmed to compensate for the losses mentioned in 4 and 5 above. The repetition of the open-ear KEMAR recording condition (1 and 3) was to ensure within-group consistency of the recordings.

The DIAA batteries, being single use, cannot be replaced or recharged and have a short shelf life once the device is unpackaged, even when the device is off. All experimentation with the DIAAs (determinations of required insertion gain and recordings) was performed within 1 month of opening, and the devices were left switched off between uses. For the digital HAs, fresh batteries were used both for the determinations of required insertion gain and at the start of the recordings.

#### Preparatory Processing

Each in-ear recording was high-pass filtered at 40 Hz with a linear-phase infinite impulse response filter in order to remove any infrasonic noise from ventilation or building vibration, which otherwise could have interfered with calibrations. Since the recordings were referenced to the end of KEMAR's meatus, they already had meatal resonances applied in passing from the loudspeaker to the meatal microphone. Consequently, the recording had to be delivered to the listener's meatus with a flat frequency response earphone. The recording was therefore linear-phase finite impulse response (FIR) filtered with the inverse frequency response of the intended delivery earphone. The desired snippet from each recording was then automatically extracted. Each snippet was written to a two-channel WAV file for presentation in the experiment proper.

A second version of the near-flat response recording, intended for use with the NH group, was produced by being steeply low-pass filtered with a linear-phase (FIR) filter with a corner frequency of 5 kHz. This was intended to act as a low-quality anchor.

## Main Experiment

### Procedure

Presentation of the recordings to each participant was performed using the PreSonus V22SL sound card driving Etymotic ER4s insert earphones. These deliver a smoother response in the meatus by bypassing possible effects of the participants' pinnae when using headphones. At the start of the experiment, the sensitivity and absolute frequency response of each transducer was checked in the KEMAR 60318-4 couplers, as well as their relative sensitivity (left/right balance). After this, calibration was rechecked just before the start of any testing session.

### Rating of Paired Comparisons

Participants were seated at a table in front of a computer screen and mouse. They were informed that they would hear sequential pairs of speech or music excerpts and that the pairs comprised the same excerpt but would likely sound different. They were instructed to answer one of the following questions after each paired presentation using a sliding scale displayed on the graphical user interface (GUI) on the screen.

- Which sound is clearer/more natural?
- Which sound is more external to your head?

The use of the phrase “clearer/more natural” arose from previous use in company marketing literature. The use of the word “external” was intended to act as a proxy for a more theoretical concept, “spaciousness.” Clarity and spaciousness are two concepts among seven previously identified by Gabrielsson (1979) as important contributors to sound quality. As it was envisaged that the concept of a sound being “external” may be difficult for participants to grasp, it was demonstrated to participants at the start of the experiment by playing a binaural recording (made in open-ear KEMAR) of a toy train circling KEMAR at ear height, along with a voice-over describing where the train was in location relative to the head.

**Figure 6.**
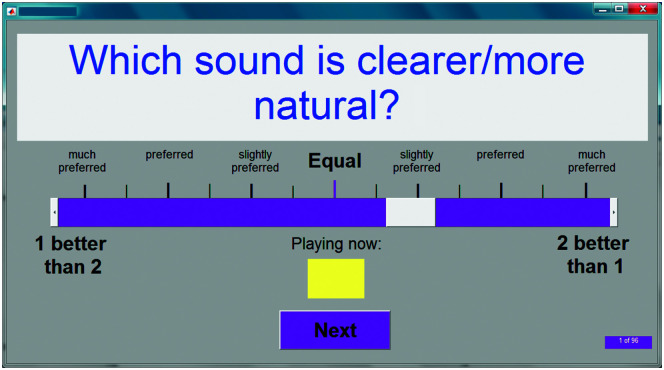
Example of the experimental graphical user interface for the collection of ratings of clarity. The gray patch on the purple horizontal bar is the slider, which the participant moved in response to the two–sound sample comparison. “1” and “2” are the labels given to each sound sample in the comparison, denoting the order in the presentation (respectively the “A” and “B” in Figures 7 and 8).

The order of presentation of blocks containing either clarity or externality trials was alternated between participants. An example of the GUI is given in Figure 6. It used a 7-point Likert scale to answer the rating questions. The software prevented participants from proceeding to the next comparison until two conditions had been satisfied.

1. They had moved the slider from its starting (neutral) position.
2. The second excerpt had played out for more than 1 s of its full length.

A block of trials included a practice run of six trials and a further 96 trials for the required paired comparisons. These were as follows:

1. NH group: 3 pairwise “device” comparisons (open-ear KEMAR, DIAA, or 5-kHz low-pass filtered DIAA) × 2 audio types (speech or music) × 2 (reversals of comparison order) × 8 trials per condition, and
2. HI group: 6 pairwise “device” comparisons (open-ear KEMAR, DIAA, ITE, or RIC) × 2 audio types (speech or music) × 2 (reversals) × 4 trials per condition.

Presentation order was randomized but counterbalanced across comparisons (e.g., “ITE vs. DIAA” and “DIAA vs. ITE”) for each participant within each block of 96 trials. Both the participants and researcher were blind as to which comparisons were playing during the experiment. The entire experiment took place within one session lasting about 2 hr, with options given for breaks from the workload.

### Derivation of a Summary Metric From the Paired Comparisons

The pattern of response in the rating data showed some participants to be very sensitive to differences between the devices and using a wide range of the rating scale, but others were much less so. A Shapiro–Wilk test showed that many of the Ratings were not normally distributed. Therefore, nonparametric statistics were performed initially using a Friedman test (a one-way nonparametric analysis of variance of the data scaled in ranks). If the result from the Friedman test was significant, then the Wilcoxon signed-ranks test was used to assess if the data point was significantly different from zero, or if the difference between pairs of data points was significantly different from zero.

**Figure 7.**
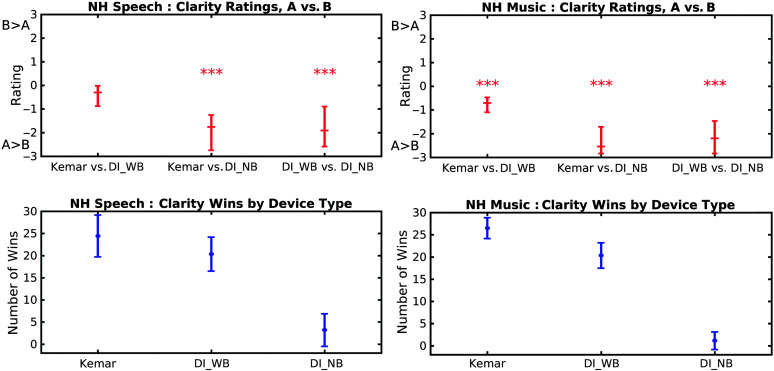
Clarity results from the normal-hearing (NH) group. The left-hand column shows results for the speech excerpt. The right-hand column shows the results for the music excerpt. In each column, the top panel shows the median rating from the aid comparisons (the error bars show interquartile ranges). Asterisks denote that there was a significant difference in the comparison (*p < .05, **p < .01, ***p < .001). A negative rating indicates a preference for the first recording condition in the “A vs. B” axis label, and a positive rating indicates a preference for the second recording condition compared. The bottom panel shows the mean number of “Wins” by device type, plotted with standard deviations. See text for further detail and the definition of a “Win.”

There were eight data sets to analyze: two types of group (NH and HI), two types of rating question (clarity and externality), and two types of audio material (speech and music). All the sets of clarity ratings given by the NH and HI groups had significant differences within their comparisons. The same pattern of results was observed across a derived measure, “Wins.” A “Win” was defined as the number of times the particular recording condition achieved a rating in excess of 0.5-scale units over its comparison recording—that is, the slider was dragged at least halfway toward “slightly preferred”; 0.5 was a semi-arbitrary choice but was based on the minimum mean difference necessary to refute the null hypothesis that the Ratings provided by the NH group (see Figure 7, top panels) were significantly different from zero using the Wilcoxon signed-ranks test. From these, a ranking was derived of the relative strengths of the comparisons. Moving from a comparison metric (Rating) to an absolute metric (Win) and performing a ranking on this allowed direct comparison between individual aids, not pairs of aids. The “Win” therefore quantified which rating differences can be deemed to be clinically “worthwhile.” When comparing across conditions within statistical tests, Bonferroni corrections were applied as appropriate.

## Results

### NH Group Results

The three recording conditions are referred to as “Kemar” for the open-ear recordings performed on the KEMAR manikin, “DI_WB” for “DIAA Wideband” (no low-pass filtering applied), and “DI_NB” for “DIAA Narrowband,” where the 5-kHz low-pass filter was applied.

#### Speech Clarity

The top left-hand panel of Figure 7 shows the rating results for speech clarity. The Friedman test on the Ratings showed a significant overall effect, *Q* = 26.8 (2*df*), *p* < .001 (denoted by the triple-asterisk marks in the panel). In detail, the Wilcoxon test showed that [Kemar vs. DI_NB] and [DI_WB vs. DI_NB] were significantly different from zero, *p* < .001, but that the third condition, [Kemar vs. DI_WB], was not significantly different from zero (*p* > .05). Comparing between the individual comparisons, the Friedman test showed that two comparisons were significantly different from each other (*p* < .001), and one was not. These differences can be converted into a rank ordering, summarized as

$$\left[\text{Kemar}\;\mathrm{vs}.\mathrm{DI}\_\mathrm{NB}\right]\approx \left[\mathrm{DI}\_\mathrm{WB}\;\mathrm{vs}.\mathrm{DI}\_\mathrm{NB}\right]>\left[\text{Kemar}\;\mathrm{vs}.\mathrm{DI}\_\mathrm{WB}\right]\text{,}$$ (1)

where “≈” denotes “is approximately equal to.”

The left-hand lower panel in Figure 7 shows the Win metric from these results. The Friedman test showed a significant overall effect, *Q* = 31.6 (2*df*), *p* < .001. Comparing across the separate aids, the Wilcoxon test showed that DI_NB was significantly different from Kemar and DI_WB (*p* ≤ .001), but that Kemar and DI_WB were not significantly different from each other (*p* > .05). A rank ordering of these differences can be summarized as

$$\left[\text{Kemar}\right]\approx \left[\mathrm{DI}\_\mathrm{WB}\right]>\left[\mathrm{DI}\_\mathrm{NB}\right].$$ (2)

#### Music Clarity

The top right-hand panel of Figure 7 shows the rating results for music clarity. The Friedman test showed a significant overall effect, *Q* = 28.9 (2*df*), *p* < .001. The Wilcoxon test showed that all three ratings were significantly different from zero, *p* < .001 (denoted by the triple-asterisk marks in the panel). Comparing between the individual comparisons, the Friedman test showed that all three ratings were significantly different from each other (*p* ≤ .022). By rank-ordering these differences, the strength of the comparison ratings can be summarized as

$$\left[\text{Kemar}\;\mathrm{vs}.\mathrm{DI}\_\mathrm{NB}\right]>\left[\mathrm{DI}\_\mathrm{WB}\;\mathrm{vs}.\mathrm{DI}\_\mathrm{NB}\right]>\left[\text{Kemar}\;\mathrm{vs}.\mathrm{DI}\_\mathrm{WB}\right].$$ (3)

The right-hand lower panel in Figure 7 shows the Win metric from these results. The Friedman test on the Wins showed a significant effect, *Q* = 35.8 (2*df*), *p* < .001. Comparing across the separate aids, a Wilcoxon test showed that all aids were significantly different from each other (*p* ≤ .001). By rank-ordering these differences, the strength of the preference can be summarized as

$$\left[\text{Kemar}\right]>\left[\mathrm{DI}\_\mathrm{WB}\right]>\left[\;\mathrm{DI}\_\mathrm{NB}\right].$$ (4)

#### Speech and Music Externality

The overall effects reported by Friedman tests were *Q* = 0.70 and 0.79 (2*df* for both) for speech and music externality, respectively, with both being insignificant, *p* > .05. Hence, externality will not be considered further with the NH group.

#### Summary of NH Results

Although there was a preference for the Kemar recordings of music over the other two recordings, this was primarily exhibited in comparison to the narrowband DIAA. The comparison between Kemar and wideband DIAA produced a less marked preference, significant for music but not for speech, but both of these ratings translated into a slightly higher number of Wins for Kemar. The narrowband DIAA recordings were easily detected by the NH group and rated very poorly, showing them to be distinctly not preferred.

### HI Group Results

The four recording conditions are referred to as “Kemar” for the open-ear recordings, “DIAA,” “ITE,” and “RIC.” No differences were observed between the responses of the N2 and N3 subgroups

#### Speech Clarity

**Figure 8.**
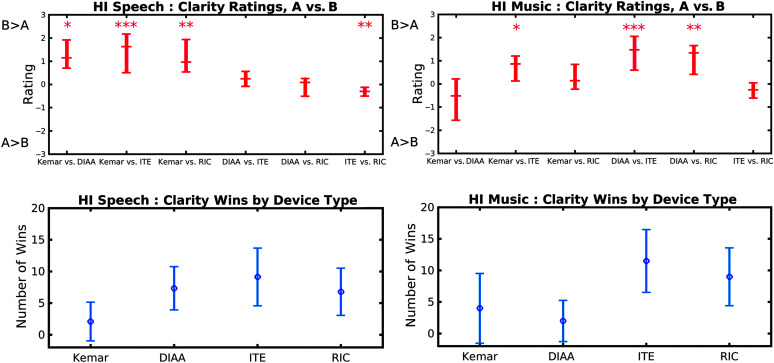
Clarity results from the hearing-impaired (HI) group for the median ratings of the aid comparisons and “Wins.” Results presented as for Figure 7. DIAA = deeply inserted analog aid device; ITE = in-the-ear hearing aid device; RIC = receiver-in-the-canal hearing aid device. *p < .05. **p < .01. ***p < .001.

The top left-hand panel of Figure 8 shows the rating results for speech clarity. The Friedman test showed a significant overall effect, *Q* = 61.9 (5*df*), *p* < .001. A Wilcoxon test showed that four ratings were significantly different from zero, *p* < .022. These are marked by asterisks (*p < .05, **p < .01, ***p < .001). The insignificant differences were [DIAA vs. ITE] and [DIAA vs. RIC] (*p* > .05). Comparing between the individual comparisons, a Wilcoxon test showed that [Kemar vs. DIAA], [Kemar vs. ITE], and [Kemar vs. RIC] ratings were significantly higher than for [DIAA vs. ITE] and [ITE vs. RIC]. (This can be summarized as the three left-hand points in the panel labeled “HI Speech: Clarity Ratings” in Figure 8 being significantly higher than the two right-hand points in the same panel.) There was a slight “in-betweenness” of the remaining point, [DIAA vs. ITE], only being significantly lower than [Kemar vs. ITE], the highest point of the left-hand triad. The ranking of these differences can be summarized as

$$\left[\text{Kemar}\;\mathrm{vs}.\;\text{DIAA}\right]\approx \left[\text{Kemar}\;\mathrm{vs}.\;\mathrm{ITE}\right]\approx \left[\text{Kemar}\;\mathrm{vs}.\;\mathrm{RIC}\right]>\left[\text{DIAA}\;\mathrm{vs}.\;\mathrm{ITE}\right]>\left[\text{DIAA}\;\mathrm{vs}.\;\mathrm{RIC}\right]\approx \left[\mathrm{ITE}\;\mathrm{vs}.\;\mathrm{RIC}\right].$$ (5)

The left-hand lower panel in Figure 8 shows the Win metric from these results. The Friedman test showed a significant effect, *Q* = 32.4 (3*df*), *p* < .001. Comparing across the conditions, a Wilcoxon test showed that Kemar was worse than the other three aids (*p* < .04) and that, although DIAA and ITE were insignificantly different from each other, RIC was worse than ITE (*p* < .001). By rank-ordering these differences, the strength of the preference can be summarized as

$$\left[\mathrm{ITE}\right]\approx \left[\text{DIAA}\right]\approx \left[\mathrm{RIC}\right]>\left[\text{Kemar}\right].$$ (6)

#### Music Clarity

The top right-hand panel of Figure 8 shows the rating results for music clarity. The Friedman test reported a significant overall effect, *Q* = 56.9 (5*df*), *p* < .001. A Wilcoxon test showed that only three of the ratings were significantly different from zero, *p* ≤ .024, [Kemar vs. ITE], [DIAA vs. ITE], and [DIAA vs. RIC] (number of asterisks denoting degree of significance as speech clarity above). Comparing between the individual comparisons, a Wilcoxon test showed that all three ratings were significantly different from the near-zero ratings (*p* < .02) and insignificantly different from each other (*p* > .05). By rank-ordering these differences, the strength of the comparison ratings can be summarized as

$$\left[\text{Kemar}\;\mathrm{vs}.\;\mathrm{ITE}\right]\approx \left[\text{DIAA}\;\mathrm{vs}.\;\mathrm{ITE}\right]\approx \left[\text{DIAA}\;\mathrm{vs}.\;\mathrm{RIC}\right]>\left[\text{Kemar}\;\mathrm{vs}.\;\text{DIAA}\right]\approx \left[\text{Kemar}\;\mathrm{vs}.\;\mathrm{RIC}\right]\approx \left[\mathrm{ITE}\;\mathrm{vs}.\;\mathrm{RIC}\right]\text{.}$$ (5)

The right-hand lower panel in Figure 8 shows the Win metric from these results. The Friedman test reported a significant overall effect, *Q* = 20.1 (3*df*), *p* < .001. Comparing across the aids, a Wilcoxon test showed that the DIAA was not significantly different from Kemar (*p* = .53) but significantly lower rated than both ITE and RIC (*p* < .018). However, Kemar was not significantly different from RIC (*p* > .05). By rank-ordering these differences, the strength of the preference can be summarized as

$$\left[\mathrm{RIC}\right]\approx \left[\mathrm{ITE}\right]>\left[\text{DIAA}\right]\approx \left[\text{Kemar}\right].$$ (8)

#### Speech and Music Externality

The overall effect for the rating of speech externality reported by the Friedman test was *Q* = 21.2 (5*df*), *p* = .001. However, a Wilcoxon test showed that only [Kemar vs. ITE] was significantly different from zero. Comparing between the comparisons, the Wilcoxon test showed that no differences were significant (*p* > .05). The rank ordering can therefore be summarized as “all comparisons insignificant.” For the music externality, the Friedman test reported *Q* = 6.46 (5*df*), *p* > .05; hence, no further consideration is given to any of the externality data for the HI group.

#### Summary of HI Results

With both speech and music, there was a general preference for the ITE and RIC recordings over those of Kemar and DIAA recordings, and for music clarity, Kemar was preferred over the DIAA. For the speech recordings, the DIAA, RIC, and ITE were ranked similarly for clarity.

## Tabulation Summary of Statistical Analyses

The rankings generated from both the Ratings and the Wins described above are shown symbolically in Table 3, the top half containing the NH data and the lower half containing the HI data. Within each half is a separation of rankings according to either comparison ratings or “Wins.” This table permits a more rapid comparison of the pattern of preferences.

**Table 3.** Summary of statistics on “Rating” and “Win” data achieving significance in the Friedman test.

Variable	Data type	Friedman *Q* (*df*)	*p*	Ranking of comparisons
(a) NH data				
Speech clarity	Ratings	26.8 (2)	< .001	[Kemar vs. DI_NB] ≈ [DI_WB vs. DI_NB] > [Kemar vs. DI_WB]
	Wins	31.6 (2)	< .001	[Kemar] ≈ [DI_WB] > [DI_NB]
Music clarity	Ratings	28.9 (2)	< .001	[Kemar vs. DI_NB] > [DI_WB vs. DI_NB] > [Kemar vs. DI_WB]
	Wins	35.8 (2)	< .001	[Kemar] > [DI_WB] > [DI_NB]
(b) HI data				
Speech clarity	Ratings	61.9 (5)	< .001	[Kemar vs. DIAA] ≈ [Kemar vs. ITE] ≈ [Kemar vs. RIC] > [DIAA vs. ITE] > [DIAA vs. RIC] ≈ [ITE vs. RIC]
	Wins	32.4 (3)	< .001	[ITE] ≈ [DIAA] ≈ [RIC] > [Kemar]
Music clarity	Ratings	56.9 (5)	< .001	[Kemar vs. ITE] ≈ [DIAA vs. ITE] ≈ [DIAA vs. RIC] > [Kemar vs. DIAA] ≈ [Kemar vs. RIC] ≈ [ITE vs. RIC]
	Wins	20.1 (3)	< .001	[RIC] ≈ [ITE] > [DIAA] ≈ [Kemar]

*Note.* “≈” denotes “is approximately equal to.” *df* = degrees of freedom; NH = normal hearing; HI = hearing impaired.

## Discussion and Conclusions

The most clear-cut results in this experiment were obtained from both listener groups in the ratings of clarity. Differences in clarity ratings were anticipated due to variations in the distortions introduced by the processing in the HAs. These (necessary) distortions can be expected to be both linear, such as a reduced bandwidth compared to the original recording, and nonlinear, since all of the aids incorporated dynamic range compression. Additionally, due to limitations in the fitting controls, there was not a perfect match between the insertion gains of the HAs when they were programmed for the HI group experiment (see Figure 2). These limitations primarily affected the high-frequency range but were also seen with the ITE where a possible vent resonance led to a +6 dB rise in the 200- to 400-Hz region. Especially for the comparison with the DIAA, this “bass boost” could have added to the clarity difference in the music excerpt.

### What Signal Features Contributed to the NH Pattern of Results?

For the NH group, the number of Wins was similar between the DIAA wideband and KEMAR. It can be assumed that this is because the DIAA largely preserved the integrity of the original open-ear recording. One contributing factor is likely to be the bandwidth of the DIAA, which we measured as a steep roll-off around 10 kHz, as well as a gentler roll-off below 300–400 Hz. When the high-frequency bandwidth was reduced (most obviously with the DIAA Narrowband recording), participants perceived a large reduction in clarity. The difference in the pattern of results between the speech and music scores (see Table 3) suggests that the wider frequency response of Kemar than that of the DIAA, particularly the low frequencies, is appreciated. We return to this point under the Clinical Implications section below.

### What Signal Features Contributed to the HI Pattern of Results?

For the HI group, the biggest contrast came from the comparison of the rating of DIAA performance against other devices for the different sound excerpts. When music was present, there was no significant difference in preferences for the DIAA or Kemar. This was despite the lack of audibility in the Kemar recordings of the higher frequencies (> 1000 Hz) where the participants' mean loss exceeded 30 dB HL. When speech was present, the lack of low frequency was less noticed, and DIAA performed near-equally with the (premium) ITE and RIC recordings. As shown in Figure 4, the near-acoustic transparency of the ITE and RIC devices permitted the direct path from the sound source to the meatus to make up for the devices' own lack of aided low-frequency response. The mean audiograms for the HI participants were less than 10 dB HL below 250 Hz, so with a higher replay level of the music, 70 dBA, the low frequencies of the music would appear to have very similar loudness to that which a person with NH would experience, hence why, for the music excerpt, the DIAA was ranked poorly. We cannot entirely rule out the influence of the inability of the DIAA to match the required N2 and N3 insertion gains as well as the ITE and RIC above the 3- to 4-kHz range. However, given the higher replay levels of the music compared to the speech and the lower high-frequency levels in the speech compared to the music, the reduced high-frequency response of the DIAA is likely to have been a small contributor to ratings. Only when a major deficiency in high-frequency levels was apparent, such as in the Kemar recordings, did a markedly lower preference appear. As with the NH results, the dynamic range control (single channel and fixed speed for the DIAA and multichannel with varying speed for the ITE and RIC), did not appear to contribute a major effect in the HI results.

### Null Results With “Externality”

The lack of effect that was found with the rating of the externality of the speech recordings is probably attributable to one or several of three possibilities.

1. Hearing impairment generally reduces the ability to binaurally process signals (e.g., Hausler et al., 1983). This tendency to a reduced spatial discrimination ability may have confounded the task (Akeroyd, 2014).
2. The lack of head movement in the experiment design, combined with use of artificial pinnae, would have led to greater front/back errors in both listener groups (Akeroyd, 2014), implying a poorer spatial resolution.
3. The medium-sized room and its low reverberation time did not give a good impression of space, but the ability to use pinna cues with the microphone position of the DIAA should allow some level of discrimination as to whether sounds were internal or external to the participant's head. In practice, participants were poor at making this distinction.

### Limitations

The following are the limitations of the study.

1. The nature of the particular DIAA device used, being a very personal device in terms of hygiene and fitting, meant that the experiment had to be structured to perform comparisons between offline recordings made on KEMAR (alternative approaches would have been both wasteful and impracticable). While this added to the flexibility of the experiment and allowed blinding of the participant and the tester, it did remove some elements of real-world usage, such as the ability to use one's own pinna cues, as well as to make head movements. It also prevented fine-tuning of the devices based on the participants' individual audiograms and real-ear responses. Another downside of this approach is that the depth of insertion achievable in a real ear (i.e., to within 4 mm of the eardrum) is not possible in the IEC 711 ear simulator coupler: It has a grille at its entrance, which limits the depth of insertion in the synthetic meatus. In effect, an insertion depth to within 10 mm of the “eardrum” was achieved. Nevertheless, this constraint is likely to have had only a small effect on the results of the experiment, as (a) the DIAAs were still sited more deeply than either the RIC or ITE devices; (b) the REOG confirmed that the XS-sized DIAA sealed the meatus adequately; and (c) the DIAA was able to approximately match the NAL-NL2 insertion gain, at least for the N2 audiogram, but less so for the N3.
2. The use of commercially available devices, combined with processing constraints used to extend battery life in the DIAA, meant that an exact match between aids in terms of processing parameters, such as compression speed, number of compression channels, insertion gain, and bandwidth, could not be achieved. The audiometric selection criteria for the HI participants, as well as the provision of two insertion responses, N2 and N3, meant that a clinically near-suitable response was available for the participants. Therefore, the results can only suggest—and not prove—which parameters were the main contributors to the effects observed. However, the results have clinical implications.

### Clinical Implications

The use of multiple two-way comparison ratings led to results that were harder to interpret clinically where a ranking of the range of devices is to be preferred. In order to account for this, our analyses included a derived measure, a “Win,” so as to generate this ranking. This measure showed that there were often minimal differences between devices, but the pattern varied with hearing impairment and with the test material, speech or music.

For those individuals who have access to a choice of HA style (e.g., completely-in-the-canal, ITE, and BTE), the eventual decision is likely to be based on numerous factors, including cosmetic appearance, usability, and sound quality. This study has demonstrated that, for those who are otologically suitable, a DIAA is a comparable option to premium digital HAs when the clarity of speech is a priority. A compromise, however, may be relatively poorer clarity of music. Theoretically, this compromise can be ameliorated by the use of open, rather than closed, fittings, but this is not possible with the DIAA due to the difficulties that would arise in removing any trapped water and the increased risk of infection.

Of the four factors outlined in the introduction that vary with the different positions of microphone and receiver in and around the ear (the degree of processing delay, accuracy of sound-field sampling, wider frequency response, and occlusion), only bandwidth appeared to be a major influence in our results. Both low and high frequencies appear to play a major role. Any or all of the remaining three factors do not appear to be major influencers. The use of venting or open fits in the ITE and RIC devices, respectively, reduces the occlusion generated by shallow insertions but has the benefit of obtaining a sufficiently extended low-frequency response for music, at least for the range of low-frequency hearing losses assessed here. As low-frequency hearing loss increases in degree beyond “mild,” then this can be expected to be less of the case.

## Data Availability Statement

The data on which these results are generated are openly available at https://www.doi.org/10.5281/zenodo.6646488. The location also includes descriptor files of the contents.
